# Characterization of Novel Integrons, In*1085* and In*1086*, and the Surrounding Genes in Plasmids from Enterobacteriaceae, and the Role for *attC*_aadA16_ Structural Features during *attI1* × *attC* Integration

**DOI:** 10.3389/fmicb.2017.01003

**Published:** 2017-06-02

**Authors:** Dongguo Wang, Wei Hou, Jiayu Chen, Linjun Yang, Zhihui Liu, Zhe Yin, Jiao Feng, Dongsheng Zhou

**Affiliations:** ^1^Department of Clinical Laboratory Medicine, Taizhou Municipal Hospital Affiliated with Taizhou University and the Institute of Molecular Diagnostics of Taizhou UniversityTaizhou, China; ^2^Department of Infection, Taizhou Municipal Hospital Affiliated with Taizhou UniversityTaizhou, China; ^3^Basic Department, Shaoxing UniversityTaizhou, China; ^4^Department of the Thyroid Gland and Breast Surgery, Taizhou Municipal Hospital Affiliated with Taizhou UniversityTaizhou, China; ^5^Department of Stomatology, Taizhou Municipal Hospital Affiliated with the Medical College of Taizhou UniversityTaizhou, China; ^6^State Key Laboratory of Pathogen and Biosecurity, Beijing Institute of Microbiology and EpidemiologyBeijing, China

**Keywords:** class 1 integron, In*1085*, In*1086*, evolutionary inferences, gene cassettes

## Abstract

Novel class 1 integrons In*1085* and In*1086*, containing the class D β-lactamase -encoding gene *bla*_OXA_, were identified in clinical enterobacterial strains. In this study, we aimed to characterize the genetic contexts of In*1085* and In*1086*, with the goal of identifying putative mechanisms of integron mobilization. Four plasmids, approximately 5.3, 5.3, 5.7, and 6.6 kb, from 71 clinical Enterobacteriaceae strains were found to contain class 1 integrons (In*37*, In*62*, In*1085*, and In*1086*, respectively). Two of these plasmids, pEco336 and pNsa292, containing In*1085* and In*1086*, respectively, were further characterized by antibiotic susceptibility testing, conjugation experiments, PCR, sequencing, and gene mapping. The OXA-type carbapenemase activities of the parental strains were also assessed. The results revealed that the novel integrons had different genetic environments, and therefore demonstrated diverse biochemical characteristics. Using evolutionary inferences based on the recombination of gene cassettes, we also identified a role for *attC*_aadA16_ structural features during *attI1* × *attC* insertion reactions. Our analysis showed that gene cassette insertions in the bottom strand of *attC*_aadA16_ in the correct orientation lead to the expression the encoded genes from the Pc promoter. Our study suggests that the genetic features harbored within the integrons are inserted in a discernable pattern, involving the stepwise and parallel evolution of class 1 integron variations under antibiotic selection pressures in a clinical setting.

## Introduction

Gene cassettes (GCs) are small, mobile elements containing one or more genes and recombination sites, and are often contained within larger genetic structures known as integrons. Several cassettes can be inserted into a single integron, where they establish a tandem array (Partridge et al., 2009). Integrons consist of three crucial components: an *intI* gene, recombination site *attI*, and a Pc promoter. *intI* encodes an integrase that mediates the integration and excision of the GCs by site-specific recombination (Barraud and Ploy, 2015). The mechanisms of integration and excision of GCs are well described, with integrations known to occur at *attI* × *attC* recombination sites (MacDonald et al., 2006; Loot et al., 2012), and excisions requiring *attC* × *attC* recombination sites, which occur in single-stranded sequences and activate the folded bottom strand (bs) (Bouvier et al., 2005, 2009). However, despite their mobility, GC rearrangements resulting in novel GC arrays rarely occur, although it is assumed that integrases could rearrange GCs, generating integron variants, under antibiotic selective pressure (Bouvier et al., 2005, 2009; Barraud and Ploy, 2015).

Generally, integrons are confirmed by the presence of *intI* and an *attI* recombination site (Barraud and Ploy, 2015). Integrons can be assigned into three classes (1, 2, and 3) based on their integrase gene sequences, with class 1 integrons most often associated with antimicrobial resistance in clinical bacterial isolates (Huang et al., 2015). By associating with transposons or being plasmid-encoded, integrons may capture genetic structures, express GCs, and mediate their own mobility despite their lack of self-mobility elements (Carattoli, 2003; Mazel, 2006). Transposon- or plasmid-associated integrons containing antibiotic resistance GCs can spread widely among Gram-negative bacteria (Barraud and Ploy, 2015). Integrons can also evolve rapidly through the acquisition, storage, and rearrangement of genes embedded in their GCs, and, by mobilization to conjugative plasmids, they play a crucial role in increasing multidrug resistance amongst clinical bacterial strains (Escudero et al., 2016). In general, integrons from clinical bacterial strains contain less than five GCs (Bennett, 2008), although integrons with up to nine antibiotic resistance genes have been reported (Naas et al., 2001). Presently, more than 130 different GCs (less than 98% identical) containing antibiotic resistance genes have been identified, along with many other GCs containing genes of unknown function (Partridge et al., 2009).

Unlike class D oxacillinases, OXA-type carbapenemase genes were not originally thought to be integrated into integrons as part of GC sequences, and were mainly shown to be encoded by chromosomal genes (Walther-Rasmussen and Høiby, 2006). However, more recent studies have shown that integrons harboring various *bla*_OXA_-type genes are present in bacterial plasmids (Wendel et al., 2016). The current study characterizes novel integrons In*1085* and In*1086* and their surrounding genes within bacterial plasmids, and explores the putative mechanism of integron mobilization.

## Materials and Methods

### Ethical Statement

This study was approved by the Ethics Committee of the Municipal Hospital of Taizhou University, Zhejiang, China, and written informed consent was obtained from each of the participants in accordance with the Declaration of Helsinki. The rights of the research subjects were protected throughout, and we confirm that this study was conducted in our hospital.

The use of human specimens and all related experimental protocols was approved by the Committee on Human Research of the indicated institutions, and was carried out in accordance with approved guidelines.

### Bacterial Strains and Susceptibility Testing

Four of seventy-one non-redundant multidrug resistant Enterobacteriaceae strains, including *Klebsiella pneumoniae* Kpn761 (harboring In*62*), non-typhoidal *Salmonella* strains Nsa243 and Nsa292 (In*37* and In*1086*, respectively), and *Enterobacter cloacae* Eco336 (In*1085*), were recovered from hospitalized patients with clinical infections. The isolates were collected between June 2013 and July 2015, and were primarily assessed for the presence of integrons by antimicrobial susceptibility testing. Bacterial species were identified by 16S rRNA gene sequencing as described previously (Frank et al., 2008). The strains harboring integrons were studied in the study. *Escherichia coli* TOP10 cells (Invitrogen, Grand Island, NY, United States) were used as a host for cloning experiments, and as a control for susceptibility testing.

The minimum inhibitory concentration (MIC) values of isolates Eco336 and Nsa292 (harboring In*1085* and In*1086*, respectively) for 12 antimicrobial agents, including cephalosporins (cefazolin, ceftazidime, and ceftriaxone), aminoglycosides (netilmicin, tobramycin, and amikacin), carbapenems (ertapenem, meropenem, and imipenem), and quinolones (norfloxacin, ofloxacin, and ciprofloxacin), were determined using the Microscan broth dilution method (Microscan, Renton, WA, United States). The MICs were interpreted in accordance with the Clinical and Laboratory Standards Institute guidelines (Clinical and Laboratory Standards Institute [CLSI], 2015).

### Plasmid Digestion with *Bam*HI, Plasmid Conjugation Experiments, Integron Cloning, and Sequencing

Plasmids from four integron-harboring strains were isolated using an AxyPrep Plasmid Miniprep kit (Axygen Biosciences, Beijing, China) according to the manufacturer’s instructions and as described previously (Wang et al., 2014). Two plasmids containing novel integrons In*1085* and In*1086* were then digested with *Bam*HI (TaKaRa, Dalian, China) and subjected to agarose gel electrophoresis to generate genetic maps.

To characterize the two novel integrons, the relevant *Bam*HI fragments of the two digested plasmids were ligated into the pMD19-T cloning vector (TaKaRa, Dalian, China). The ligation mixtures were electroporated into *E. coli* TOP10 cells, which were then plated on LB medium with medium supplemented with ampicillin (100 μg/mL) and incubated over-night at 37°C. Plasmids from any resulting *aacA4*-positive colonies were isolated by PCR amplification and sequencing (Supplementary Table S1), and the inserts were amplified using the primers specified in Supplementary Table S1 and the following thermal cycler conditions: 3 min at 94°C, 30 cycles of 1 min each at 94, 54, and 72°C, followed by 10 min at 72°C. The total reaction volume was 25 μL, and the total eluent volume was 10 μL. Following amplification, the amplicons were separated by gel electrophoresis on a 0.6% agarose gel run at 90 V for 90 min in 0.5 × TBE buffer. Fragments corresponding to plasmid DNA and integrons were recovered, and the initial positions of the relevant genes in the recombinant plasmids were determined according to a previously established method for estimating plasmid DNA sizes (Wang et al., 2003). Thus, the sequences of both integrons, including upstream and downstream genes, were obtained, by PCR amplification and sequencing (Supplementary Table S1), and the genetic structures were mapped and characterized by next generation sequence annotation and genome comparison. Plasmids harboring In*1085* and In*1086*, In*1085*-TOP10 and In*1086*-TOP10 were studied, and the values of relevent susceptibility testing were confirmed (**Table 1**) by conjugation experiments in according with previous report (Wang et al., 2014).

**Table 1:** Antimicrobial drug susceptibility profiles.

Antibiotics	MIC (mg/L)/antimicrobial susceptibility				
	
	In*1085*	In*1086*	In*1085*-TOP10	In*1086*-TOP10	TOP10
**Cephalosporins**					
Cefazolin	128/R	128/R	16/R	16/R	1/S
Ceftazidime	64/R	128/R	4/R	8/R	0.5/S
Ceftriaxone	64/R	64/R	8/R	8/R	0.5/S
**Carbapenems**					
Ertapenem	16/R	16/R	8/R	8/R	0.5/S
Meropenem	16/R	16/R	8/R	8/R	0.5/S
Imipenem	8/R	8/R	4/R	4/R	0.25/S
**Aminoglycosides**					
Netilmicin	128/R	256/R	16/R	32/R	2/S
Tobramycin	128/R	256/R	32/R	32/R	0.025/S
Amikacin	256/R	512/R	32/R	64/R	1/S
**Fluoroquinolones**					
Norfloxacin	0.10/S	0.10/S	0.10/S	0.05/S	0.05/S
Ofloxacin	0.005/S	0.005/S	0.005/S	0.003/S	0.003/S
Ciprofloxacin	0.25/S	0.25/S	0.25/S	0.125/S	0.0125/S


### Sequence Annotation and Genome Comparison

Open reading frames (ORFs) were predicted using RAST^1^, and further annotated using BLASTP and BLASTN searches^2^ against the UniProtKB/Swiss-Prot^3^, and National Center for Biotechnology Information non-redundant^4^ databases. Annotation of drug resistance genes, mobile elements, and other genes was based on CARD analysis^5^, the β-lactamase database^6^, ISfinder^7^, and INTEGRALL^8^. Sequence comparisons were performed using BLASTN and CLUSTALW2^9^. Gene organization diagrams were drawn using Inkscape^10^.

### Detection of Carbapenemase Activity

The presence of classes A, B, and D carbapenemase activity in cell extracts from non-typhoidal *Salmonella* strain Nsa292 and *E. cloacae* strain Eco336 was determined using a modified CarbaNP test (Chen et al., 2015). Briefly, overnight cultures of each strain grown in Mueller-Hinton (MH) broth were diluted 1:100 into 3 mL of fresh MH broth, and then incubated at 37°C with shaking at 200 rpm to an optical density at 200 nm of 1.0–1.4. When required, ampicillin was used at 200 μg/mL. Bacterial cells were harvested from 2 mL of culture, and cell pellets were washed twice with 20 mM Tris-HCl (pH 7.8). Each cell pellet was resuspended in 500 μL of 20 mM Tris-HCl (pH 7.8), lysed by sonication, and centrifuged at 10,000 × *g* for 5 min at 4°C. Each 50-μL supernatant (containing the enzymatic bacterial suspension fraction) was mixed with 50 μL of the following substrates (I to V), followed by incubation at 37°C for a maximum of 2 h: substrate I: 0.054% phenol red and 0.1 mM ZnSO_4_ (pH 7.8); substrate II: 0.054% phenol red, 0.1 mM ZnSO_4_ (pH 7.8), and 0.6 mg/μL imipenem; substrate III: 0.054% phenol red, 0.1 mM ZnSO_4_ (pH 7.8), 0.6 mg/μL imipenem, and 0.8 mg/μL tazobactam; substrate IV: 0.054% phenol red, 0.1 mM ZnSO_4_ (pH 7.8), 0.6 mg/μL imipenem, and 3 mM EDTA (pH 7.8); and substrate V: 0.054% phenol red, 0.1 mM ZnSO_4_ (pH 7.8), 0.6 mg/μL imipenem, 0.8 mg/μL tazobactam, and 3 mM EDTA (pH 7.8).

### Nucleotide Sequence Accession Numbers

The sequences of novel integrons In*1085* and In*1086* were deposited in the GenBank database under accession numbers KP870111 and KP870112, respectively.

## Results and Discussion

### Integron Cloning Experiments and Antibiotic Susceptibility Testing

Four strains, including *K. pneumoniae* strain Kpn761 (harboring In*62*), non-typhoidal *Salmonella* strains Nsa292 and Nsa243 (In*1086* and In*37*), and *E. cloacae* strain Eco336 (In*1085*), were selected for further study. Non-typhoidal *Salmonella* Nsa292 and *E. cloacae* Eco336 showed resistance to aminoglycoside, cephalosporin, and carbapenem antibiotics (**Table 1**), while the remaining two strains were only used for reference in this study and will be described elsewhere. *E. cloacae* Eco336 was isolated from the urine of a urological surgery patient, *K. pneumoniae* Kpn761 (In*62*) was isolated from a blood culture of an intensive care unit patient, and non-typhoidal *Salmonella* strains Nsa292 and Nsa243 (In*37*) were isolated from blood cultures of patients hospitalized with infections.

**FIGURE 1:**
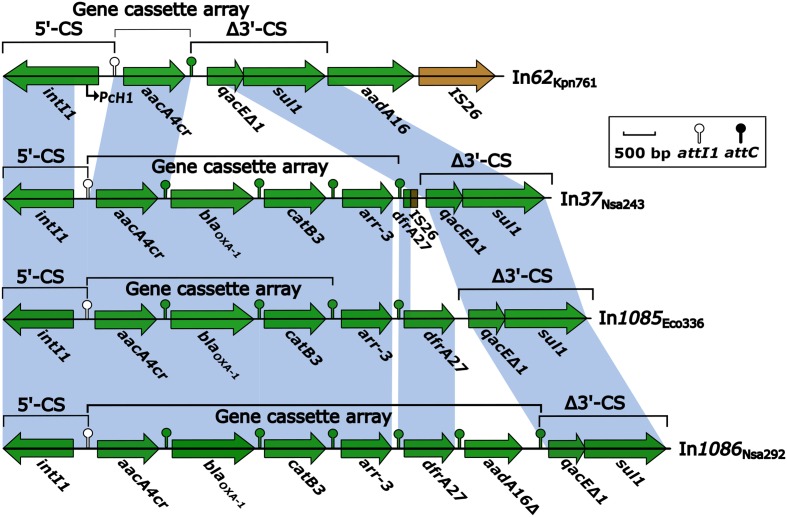
Comparison of integron structures based on *Bam*HI digestion of plasmids. Genes are denoted by arrows, and are colored based on gene function classifications. Shaded areas denote regions of homology with >97% nucleotide sequence identity. Only one gene cassette (GC) was identified in In*62*_Kpn761_ (In*62*), as the *aadA16* sequence was located beyond the integron. Four GCs were identified in In*37*, as the remnant of *dfrA27* was non-functional. Three functional GCs were identified in In*1085*, with both GC*arr-3* and GC*dfrA27* deemed non-functional. Six GCs were identified in In*1086*, although GC*aadA16* was truncated, and the *attC* sequence was mutated. In*37* and In*62* sequences have been deposited in GenBank under the accession numbers KR338349 and KR338350, respectively, and will be described in more detail in another study.

Following *Bam*HI digestion and ligation into a pMD19-T cloning vector, the four integron-containing recombinant plasmids were transformed into *E. coli* TOP10 cells. Antibiotic susceptibility test results for the resulting TOP10 cells containing the recombinant plasmids are listed in **Table 1**. The recombinant plasmids were also electrophoresed to estimate their sizes. The susceptibility test results also indicated that the conjugation experiments were successful (**Table 1**), and that the observed antibiotic resistance was conferred by plasmid-mediated genes. The electrophoresis results following *Bam*HI digestion indicated that the sizes of the In*37*, In*62*, In*1085*, and In*1086* integrons were approximately 5.3, 5.3, 5.7, and 6.6 kb, respectively. Usually, class 1 integrons that integrate within transposons or are encoded on plasmids display regular mobilization and transformation capabilities (Carattoli, 2003; Mazel, 2006). As such, integrons can change from one type to another, with the possibility of generating novel types (**Figure 1**).

### Carbapenemase Activities, Genetic Features, and Environments of Integrons

Both transformant strains containing the novel integrons, In*1085*-TOP10 and In*1086*-TOP10, demonstrated class D carbapenemase activity (data not shown), showing resistance to cephalosporins, carbapenems, and aminoglycosides. However, both strains were susceptible to fluoroquinolones (**Table 1**). Of the four integrons, In*62* apparently represents the most primitive form. It carried two different resistance markers, but only one single-gene GC (GC*aacA4cr*) (**Figure 1**). In contrast, In*37*, In*1085*, and In*1086* contained four or five GCs (**Figure 1**), containing determinants of at least three different classes of antibiotics. These three larger integrons most likely confer the multidrug resistance phenotype that was demonstrated in the results of antibiotic susceptibility testing of both In*1085* and In*1086*. Interestingly, In*37* was almost identical to novel integron In*1085*, except for the lack of GC*drfA27*, which was replaced by non-functional remnants of *drfA27* and IS*26* in In*37* (**Figure 1**). In addition, a hybrid *attC*, *attC*_aar-3/catB3_, was identified downstream of the GC*arr-3* in In*1085*. This type of hybrid *attC* is unlikely to be functional for recombination of the GC (GC*arr-3*). And, instead of the usual 5′-untranslated region (UTR) upstream of the GC*dfrA27* in In*1085*, the upstream *attC* matched the 5′-UTR of GC*arr-3*, while the downstream *attC* was deleted. Therefore, neither GC*dfrA27* nor GC*arr-3* in In*1085* appear to show any integron features, and are likely to be pseudo-integrons. However, the remaining three GCs (GC*aacA4cr*, GC*bla*_OXA-1_, and GC*catB3*) do appear to be functional.

In*1086* differed from In*1085* by the presence of an extra GC, GC*aadA16* (**Figure 1**). Noticeably, GC*aadA16* appeared to be truncated, and was thus named GC*aadA16*Δ. The *attC* of GC*aadA16* also appeared to be mutated to *attC*_qacE/aadA16_, rather than the normal *attC*_aadA16_ sequence. However, the mutation did not result in any changes in biochemical properties. Thus, In*1086* was characterized as containing six GCs. Neither of the novel integrons contained Pc variant sites or 19-bp ORF11 duplicates, but did contain non-functional remnants of *intI1* ORFs.

**FIGURE 2:**
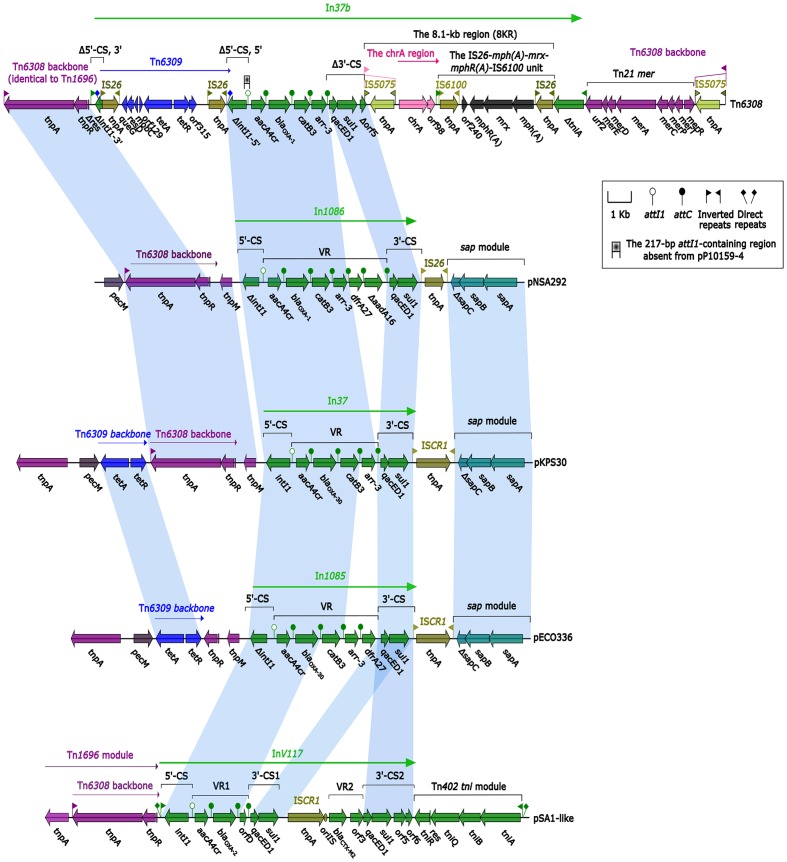
Comparison of plasmids and transposons. Genes are denoted by arrows and are colored based on gene function classifications. Shaded areas denote regions of homology with >97% nucleotide sequence identity. Tn*6308* was described by Sun et al. (2016), pKPS30 refers to the partial sequence of plasmid KPS30 described by Compain et al. (2014), while pSA1-like shows similarity to pSA1 described by Soler Bistué et al. (2006). pNSA292 and pECO336 are partial plasmids from non-typhoidal *Salmonella* strain Nsa292 and *Enterobacter cloacae* strain Eco336, harboring novel integrons In*1085* and In*1086*, respectively.

Sequence comparisons revealed a high degree of homology at nucleotide level between Tn*6308* (Sun et al., 2016) and pNSA292 (harboring In*1085*), and between pSA1-like (Soler Bistué et al., 2006) and pECO336 (harboring In*1086*) (**Figure 2**). Even greater homology was observed between pNSA292 and pECO336 and pKPS30 (Compain et al., 2014) (**Figure 2**). pNSA292 shared identity with Tn*6308* in both integron regions, except for a lack of GC*dfrA27* and GCΔ*aadA16* in Tn*6308*, and was confirmed by the high degree of sequence identity between the backbones of Tn*6308* and pNSA292 upstream of both integron regions, and at the *sap* module (**Figure 2**). pECO336 also showed partial homology to pSA1-like in the integron regions, and to the Tn*6309* backbone and *sap* module in the up- and down-stream integron regions (**Figure 2**). However, both pNSA292 and pECO336 showed greater homology to pKPS30 in both integron regions (**Figure 2**).

However, there were a few structural differences between pNSA292 and pECO336, and thus, the plasmids showed different biochemical characteristics. Because of the mobility of transposons and plasmids, we hypothesize that novel integrons In*1085* and In*1086*, located on pEco336 and pNsa292, respectively, have the potential to mobilize. In addition, these integrons may be related to other plasmids and transposons such as Tn*6308*, Tn*6309*, Tn*1696*, pKPS30, and pSA1-like (**Figure 2**).

### Evolutionary Inferences for the Recombination of GCs

**FIGURE 3:**
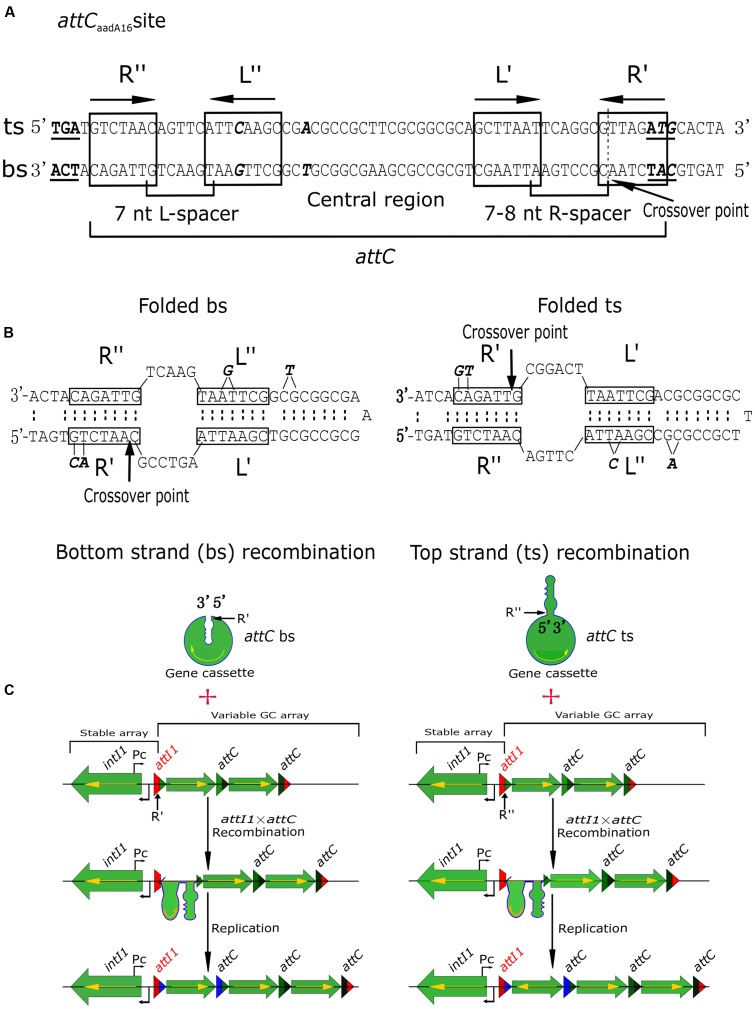
Schematic diagrams showing recombination of GCs. **(A)** Architecture of *attC*_aadA16_. The start and stop codons on the top strand (ts) and bottom strand (bs) are in bold and are underlined. Core sites are boxed and labeled, with the end points of the spacers indicated, and the extrahelical bases on the bs and ts shown in bold. The position of the GC “crossover point” is shown as a dotted line, and indicated by an arrow. **(B)** Folded bs and ts recombinations for the *attC*_aadA16_ site showing the deletion of the extrahelical bases. **(C)** If recombination takes place in the bs of the *attC* site (R′) and in the bs of *attI1*, the encoded gene can be expressed from the Pc promoter. However, if recombination occurs in the ts of the *attC* site (R″) and in the bs of *attI1*, the GC is inserted in the opposite orientation (yellow arrow) and no gene expression can occur from the Pc promoter.

Recombination at specific *intI1* sites is highly regulated, thereby differing from other reactions mediated by tyrosine recombinases, while recombination at *attC* sites is much more complicated and variable (Partridge et al., 2009). *attC*_aadA16_ comprises two simple sites, each consisting of a pair of conserved “core sites” (7 or 8 bp), designated R″/L″ and L′/R′ (Recchia and Sherratt, 2002; Escudero et al., 2016). R″/L″ sites are separated by a 7-bp spacer, and L′/R′ by a 7- or 8-bp spacer (**Figure 3A**). R″/L″ and L′/R′ pairs are separated by a central region (**Figure 3A**). Usually, R″/L″ and L′/R′ are reverse complements of each other, with R″/R′ and L″/L′ generally being complementary, except for the removal of an extra base in the L″ site [G for a bs array and C for a top strand (ts) array] and two bases in the R′ site (A and C for a bs array and T and G for a ts array) (**Figure 3B**, in bold and italic). The central region is usually a defective inverted repeat, unless there has been a deletion of a single T (bs array) or A (ts array) nucleotide (**Figure 3B**, in bold and italic). There are two regions in class 1 integrons: stable arrays, including *intI1* and the beginning of the *attI1* sequence, and variable arrays, involving the end of the *attI1* sequence and the GCs. GC integrations take place at *attI* × *attC* recombination sites (**Figure 3C**), and it is essential that the complete coding sequence of the GC is expressed from the promoter, Pc (**Figure 3C**). If the GC is inserted in the opposite orientation, it will not be expressed (**Figure 3C**). Only a few integrons have been shown to contain the Pc promoter (Bissonnette et al., 1991; Tolmasky and Crosa, 1993; Biskri and Mazel, 2003; Szekeres et al., 2007; da Fonseca and Vicente, 2012; Guerout et al., 2013), with most GCs, including the stable arrays within novel integrons In*1085* and In*1086*, appearing to be promoterless (**Figures 2**, **3**). Integration of GCs in the opposite orientation would likely hamper the rapid adaptation of integrons (Nivina et al., 2016). Correct orientation of the GCs confirms the selectivity of the integrase toward the bs (**Figure 3C**) (Nivina et al., 2016). Functional integration does not follow ts recombination, especially if the ts of *attC* is presented as a substrate (Nivina et al., 2016), because the *attC* sequence is not conserved, and the interaction between *IntI* and the folded *attC* is mostly non-specific (MacDonald et al., 2006). Hence, specific recognition of the bs is the main determinant of successful GC integration. Therefore, determinants required for specific recognition of the bs are likely to be present in the structural features of the variable GC array (**Figure 3C**). These results can help in determining the formation of integrons from clinical sources.

## Conclusion

Novel integrons In*1085* and In*1086*, located on plasmids from clinical bacterial isolates, showed different gene environments. Because of these variations in genetic structure, the two integrons displayed different biochemical characteristics. Using evolutionary inferences for the recombination of the GCs, we also determined that *attC*_aadA16_ plays an important role during *attI1* × *attC* insertion reactions, causing preferential recombination of the bs and ensuring that cassettes are inserted in the correct orientation. These findings are evidence of the stepwise and parallel evolution of integrons under antibiotic selection pressures present in clinical settings.

## Author Contributions

Conception and design of the study: DW. Acquisition of data: DW, WH, JC, DZ, LY, ZY, JF, and ZL. Analysis and interpretation of data: DW, WH, and DZ. Drafting the article: DW. Critical revision: DW, WH, JC, LY, DZ, ZY, JF, and ZL. All authors read and approved the final manuscript.

## Conflict of Interest Statement

The authors declare that the research was conducted in the absence of any commercial or financial relationships that could be construed as a potential conflict of interest.

## Footnotes

1 http://rast.nmpdr.org/

2 https://blast.ncbi.nlm.nih.gov/Blast.cgi

3 http://web.expasy.org/docs/swiss-prot_guideline.html

4 https://www.ncbi.nlm.nih.gov/

5 http://arpcard.mcmaster.ca

6 http://www.ncbi.nlm.nih.gov/pathogens/submit_beta_lactamase

7 https://www-is.biotoul.fr/

8 http://integrall.bio.ua.pt/?

9 http://www.ebi.ac.uk/Tools/msa/clustalw2/

10 https://inkscape.org
