# Supplementary material for: Characterization of Novel Integrons, In1085 and In1086, and the Surrounding Genes in Plasmids from Enterobacteriaceae, and the Role for attCaadA16 Structural Features during attI1 × attC Integration
Source: Front Microbiol. 2017 Jun 2;8:1003. doi: 10.3389/fmicb.2017.01003 (PMC5454043; doi:10.3389/fmicb.2017.01003)
Supplement: Supplementary file 2 [file Data_Sheet_2.doc]

**Supplementary file**

| Primer | | Primer sequence (5’-3’) | Target length (bp) | Reference |
| --- | --- | --- | --- | --- |
| *sapABC* | F | TTACCCATTGGTTGTCG | 420 | Lupp et al., 2002 |
|  | R | GGATCCTGGAAAATCAT |  |  |
| *tetA* | F | GTGAAACCCAACATACCCC’ | 888 |  |
|  | R | GAAGGCAAGCAGGATGTAG |  |  |
| *tnpM* | F | TCAACCTGACGGCGGCGA | 348 |  |
|  | R | GGAGGTGGTAGCCGAGG |  |  |
| *tetR* | F | CCAGTTTGCGTGTCGTCAGA | 651 |  |
| *tetA* | R | CGGCAGGCAGAGCAAGTAGA | 1200 |  |
| *pecM* | F | AGGACGCCGATGATTTGAAG | 885 |  |
|  | R | GCGGGTTTATTGCTCGTGAT |  |  |
| tnpR | F | FTCTTCGCAACACGCACCA | 1834 |  |
|  | R | ACCTCGCCGTGGAAATAG |  |  |
| *tnpA1* | F | ATCCGCATCGGGAAAGCC | 1144 |  |
|  | R | CTGCTCGTCGGCAAAGGA |  |  |
| *tnpA2* | F | GCCAAGACACTGCTGCCTAA | 1082 |  |
|  | R | TTGACGGAAGCGAAACACG |  |  |
| *tnpA3* | F | CAAGTTTCTGTCGGCCTTCA | 1434 |  |
|  | R | CACGCCTTTGCTCCTGGGT |  |  |
| *tnpA* | F | ATACGCCATTCGCCTCAG | 1320 |  |
|  | R | GTCGGCAAGGTGGTCTCA |  |  |
| 5'-CS | F | GGCATCCAAGCAGCAAGC | Variable |  |
| 3'-CS | R | AAGCAGACTTGACCTGAT |  |  |
| *Int1* | F | AGCACCTTGCCGTAGAAGAACAG | 3,500 | This study |
|  | R | GTCATAATCGGTTATGGCATCGC |  |  |
| *intI1* | F | GGGTCAAGGATCTGGATTTCG | 1,250 |  |
|  | R | ACATGCGTGTAAATCATCGTCG |  |  |
| *qacE1*-*sul1* | F | ATCGCAATAGTTGGCGAAGTGCA  AGGCGGAAACCCGCGCC | 797 |  |
| VR | R | GGCATCCAAGCAGCAAGCAAGC  AGACTTGACCTGAT | Variable |  |

**TABLE S1 Primers used for PCR amplification and sequencing**
